# Sensitization to salmon among occupationally exposed Norwegian salmon processing workers: identification of IgE-reactive proteins

**DOI:** 10.3389/falgy.2026.1735903

**Published:** 2026-02-16

**Authors:** Inga Elda, Miriam Grgic, Gro Tjalvin, Carl Fredrik Fagernæs, Anje Christina Höper, Kaja Irgens-Hansen, Hilde Brun Lauritzen, Berit Bang

**Affiliations:** 1 Department of Occupational and Environmental Medicine, University Hospital North Norway, Tromsø, Norway; 2 Department of Medical Biology, UiT The Arctic University of Norway Faculty of Health Sciences, Tromsø, Norway; 3 Department of Global Public Health and Primary Care, University of Bergen Faculty of Medicine, Bergen, Norway; 4 Department of Occupational Medicine, Haukeland University Hospital, Bergen, Norway; 5 Department of Occupational Medicine, St Olavs Hospital Trondheim University Hospital, Trondheim, Norway; 6 Department of Public Health and Nursing, Faculty of Medicine and Health Sciences, Norwegian University of Science and Technology, Trondheim, Norway; 7 Department of Community Medicine, UiT The Arctic University of Norway Faculty of Health Sciences, Tromsø, Norway

**Keywords:** occupational allergens, occupational allergy, salmon allergy, salmon processing, thermolabile allergens

## Abstract

**Introduction:**

Salmon processing workers are exposed to bioaerosols and are at risk of developing respiratory diseases and other hypersensitivity reactions. The aim of this study was to investigate the prevalence of allergic sensitization to salmon in a study population of Norwegian salmon processing workers and to investigate salmon proteins involved in IgE-binding.

**Method:**

A total of 977 salmon processing workers were tested with skin prick test (SPT) using both in-house salmon extracts, commercial extracts from cod and salmon, and specific IgE (sIgE) to Atlantic salmon (*Salmo salar*). They were also invited to answer a general questionnaire, including questions on asthma, work-related symptoms, and food-allergy to salmon. Serum from 71 sensitized workers with either a positive SPT and/or elevated sIgE to salmon, were further analyzed by immunoblot, with in-house and commercial protein extracts. Salmon proteins which were most frequently involved in IgE-binding were identified using mass spectrometric analyses of SDS-PAGE protein bands.

**Results:**

We determined a prevalence of allergic sensitization to salmon of 7.3% (*n* = 71) in the present study population. Fifty-six workers had at least one positive SPT, with most having a reaction to the in-house raw muscle extract (61%), followed by in-house mucus (42%), in-house cooked muscle (17%), commercial cod (11%), commercial salmon (8%), and in-house skin (3%). All sensitized workers had IgE-binding to proteins in at least one of the protein extracts, with immunoblot protocols: mucus (100%), raw muscle (79%), cooked muscle (20%), skin (6%), and commercial cod (24%). Most frequent IgE-binding was seen in the 60–70 and >131 kDa area for mucus, and 60–70 kDa for raw muscle. Work-related symptoms were reported by 43 workers. Only three workers had self-reported allergy to salmon related to food intake, whereas 10 workers had self-reported doctor-diagnosed asthma. With mass spectrometry, known allergens were identified, as well as potentially novel allergens with possible clinical relevance.

**Conclusion:**

Norwegian salmon processing workers are exposed to various salmon tissues at work, containing proteins which might cause allergic sensitization. Allergens other than the major fish allergen parvalbumin, including allergens not previously identified as salmon allergens, seem to play an important role in the occupational setting.

## 1 Introduction

Norway is recognized as the world's leading producer and exporter of Atlantic salmon (*Salmo salar*) (1). Previous studies have shown that workers within this industry are exposed to bioaerosols containing biologically active molecules such as allergens and enzymes (2–5). Currently, there are seven registered allergens for Atlantic salmon in the WHO/IUIS allergen nomenclature database: beta-parvalbumin 1, beta-enolase, aldolase A, tropomyosin, collagen alpha, creatine kinase, and triosephosphate isomerase (6).

Parvalbumin is recognized as the major fish allergen and was first characterized as an allergen in cod (7). It is a Ca^2+^-binding muscle protein, with high stability towards thermal treatment and other forms of processing (8) and exhibits a highly cross-reactive nature (9). The protein has been registered as an allergen in several fish species (10–14). Enolase and aldolase are muscle proteins involved in the glycolytic pathway, initially identified as allergens in cod, salmon, and tuna among patients allergic to fish, some of which were not sensitized to parvalbumin (12). Enolase and aldolase are thermolabile proteins (12), meaning that their biological function is not retained upon exposure to high temperatures. Hence, they could be more relevant as possible sensitizers when handling raw fish, such as in the salmon processing industry, compared to exposure through ingestion in which the fish is usually processed. Tropomyosin is known as the major allergen in crustaceans (15) but is also recognized as a fish allergen (16). Collagen is a structural protein of the extracellular matrix, first identified as an allergen in fish-allergic patients showing IgE-binding to collagen in a pooled protein extract from salmon, barramundi, and yellowfin tuna (17). A later study among food-allergic patients from Hong Kong and Japan found collagen to be the second most common protein next to parvalbumin to cause allergic sensitization (18). Creatine kinase, a heat-labile protein (19), has been described as an important allergen in the occupational context, with a case-report describing occupational asthma and urticaria in a fishmonger sensitized to this allergen (20). Triosephosphate isomerase, in addition to being a registered salmon allergen (14), is also a recognized crustacean (21) and mite (22) allergen. In addition, the three salmon proteins glyceraldehyde-3-phosphate dehydrogenase (GAPDH), pyruvate kinase, and glucose-6-phosphate isomerase (G-6-PI) have been described as IgE-binding proteins in a previous study among fish-allergic patients (14).

There is a need for updated knowledge on sensitization and work-related symptoms among salmon processing workers. The present study is part of the Norwegian multicenter study SHInE: Effects of Interventions to Prevent Work-Related Asthma, Allergy, and Other Hypersensitivity Reactions in Norwegian Salmon Industry Workers (23), investigating bioaerosol exposure and health outcomes as well as interventions that could possibly reduce bioaerosol exposure in Norwegian salmon processing facilities. A recent publication with data from the study reported a 20% prevalence of work-related asthma symptoms among workers exposed to bioaerosols from salmon (24).

In a European Association of Allergy & Immunology (EAACI) position paper, authors highlighted the need for more research on occupational respiratory food allergy and the role of airborne primary sensitizers in this form of allergy (25). During salmon processing, workers are exposed to parts of the fish that are commonly removed before eating, such as mucus and skin. Consequently, they might be exposed to allergens that are not relevant for seafood ingestion. Furthermore, the material that workers are exposed to is raw, which might make thermolabile allergens such as e.g., aldolase, enolase, and creatine kinase more important in occupational allergic sensitization. We have previously reported on the presence of parvalbumin, enolase, aldolase, and collagen in protein extracts from different salmon tissues: raw and cooked muscle, outer mucus coating, and skin (26). In the present study, we present data on the prevalence of sensitization to salmon in a population of 977 salmon processing workers. The frequency and relative strength of IgE-binding to known and possibly novel salmon allergens are reported, as well as work-related symptoms from upper and lower airways, and eyes.

## 2 Materials and methods

### 2.1 Study population

The present study is part of the Norwegian multicenter study SHInE (23). Between September 2021 and June 2023, salmon processing workers from nine different salmon processing plants along the Norwegian coastline were invited to participate in the study. A detailed description of the plants is available in a recent paper from the study (27). A total of 977 workers underwent occupationally relevant health examinations and 71 were included in this study based on the following criteria: 1) positive SPT (wheal diameter ≥3 mm larger than the negative control) to at least one of the following extracts: in-house raw and cooked muscle, mucus and skin, and commercial cod and salmon; and/or 2) positive sIgE to salmon (≥0.1 kU/L, ImmunoCAP, Phadia AB, Uppsala, Sweden) using workers’ sera. For workers that had participated more than once due to the structure of the SHInE study (23), only data from the first collection was included in this study.

### 2.2 Questionnaire

The participants were invited to answer a general questionnaire, including questions about age, gender, self-reported doctor-diagnosed asthma, allergic symptoms following salmon ingestion, smoking history, and work-related symptoms during the past week. The question on work-related symptoms was adapted from a previously validated instrument (28), and included items on upper and lower respiratory, and eye symptoms. Work-related symptoms were defined by the question: “*During the past week, have you experienced any of the following symptoms during your working day? Grade the symptoms (circle the appropriate number) on a scale from 0 (no symptoms) to 4 (severe symptoms)”.* The question was further divided into the following subcategories: 1) eye symptoms: “*itching”*, “*swelling”* and “*discharge”*, 2) nose symptoms: “*itching”*, “*sensation of fullness, congestion, or blockage”*, and “*sneezing”* and “*discharge or runny nose”*, and 3) respiratory symptoms: “*cough”*, “*wheeze (whistling in your chest)”*, “*mucus which is difficult to cough up”*, and “*shortness of breath or chest tightness”*. The answers were interpreted in such a way that if a participant had circled 1 or higher on at least one of the symptoms under each subcategory, that was defined as a “yes” for that subcategory. Thus, the work-related symptoms are presented as eye, nose, and lower respiratory. Self-reported allergy to salmon was considered positive if workers answered “yes” to the question: “*Have you ever been ill or experienced symptoms within two hours of eating salmon? (e.g., rash, itching, diarrhea, vomiting, runny or stuffy nose, wheezing)”.* Asthma was defined as self-reported doctor-diagnosed asthma by answering “yes” to: “*Do you have, or have you ever had asthma?”* and “*Have you ever had asthma diagnosed by a doctor?”*.

### 2.3 Serum analysis

Blood samples from workers participating in health examinations were collected during field work and appropriately stored before further analysis. Serum was analyzed for total and specific IgE at the Department of Laboratory Medicine at the University Hospital of North Norway, using a fluorescence enzyme immunoassay (FEIA) with the Phadia ImmunoCAP™ (Thermo Fisher Scientific, Uppsala, Sweden). Workers were considered atopic if they had sIgE ≥0.35 kU/L to at least one of the two common inhalation panels: Seasonal IP6 (birch, timothy, mugwort, Alternaria alternata, Cladosporium herbarum) and perennial IP7 (cat, horse, dog, pteronyssinus, rabbit). Elevated sIgE to salmon (f41) was defined as sIgE ≥0.1 kU/L (29).

### 2.4 Skin prick test

Skin prick testing (SPT) was done by trained clinical personnel during the field work. Extracts used were commercial cod (ALK-Abelló Nordic, Hoersholm, Denmark) and salmon (Lofarma, S.p.A, Milan, Italy), as well as in-house protein extracts from salmon: raw and cooked muscle, outer mucus coating, and skin. The in-house salmon extracts were produced as previously described in detail, including in-house negative controls (26). Briefly, proteins from raw and cooked muscle, outer mucus coating, and skin were extracted with different extraction buffers adjusted to the tissue types. The commercial positive and negative controls were 1% histamine, and 0.9% saline, respectively (Soluprick, ALK-Abelló Nordic, Hoersholm, Denmark). The SPT was performed by placing droplets (15 μl) of the extracts on the volar forearm, which was then pricked into the skin by sterile lancets. Reactions were read after 15 min. The tests were considered positive if the wheal diameter on the skin was ≥3 mm larger than the negative control.

### 2.5 SDS-PAGE and immunoblotting

The materials and chemicals used in the following section were all of analytical grade. Ten μl of in-house salmon protein extracts, as well as commercial extracts from cod and salmon, were separated electrophoretically on a NuPAGE 4%–12% Bis-Tris gel (Invitrogen, Thermo Fisher Scientific, Carlsbad, CA, USA). Proteins were then transferred to a nitrocellulose membrane using Power Blotter—Semi-dry Transfer System (Invitrogen, Thermo Fisher Scientific, USA). Successful transfer was confirmed by staining the membrane with Ponceau S (Thermo Scientific, Waltham, MA, USA). Subsequently, the membrane was blocked for 60 min in blocking buffer (4% milk powder, 0.05% Tween® 20). Following blocking, the membrane was incubated with worker's sera diluted in blocking buffer (1:25) for 24 h at 4 °C, shaking. On the following day, immunoblotting using the iBind Western Automated system (Invitrogen, Thermo Fisher Scientific, Carlsbad, CA, USA) was done, following manufacturers’ protocol. Briefly, the membrane was immersed in 1× iBind Flex™ Solution (Invitrogen, Thermo Fisher Scientific, Carlsbad, CA, USA), before it was placed protein-side down on the iBind™ Flex Card (Invitrogen, Thermo Fisher Scientific, Carlsbad, CA, USA). The diluted antibodies and the buffer were added to the wells before the cover was closed. The primary antibody used was monoclonal mouse anti-IgE (Novus Biologicals, Littleton, CO, USA) diluted 1:2000, and the secondary antibody was goat anti-mouse IgG HRP conjugate (Bio-Rad, Hercules, CA, USA) diluted 1:4000. Both antibodies were diluted using the 1× iBind Flex™ Solution. The immunoblot run was performed for 4 h at room temperature prior to development of the membranes using Supersignal™ West Femto Maximum Sensitivity Substrate kit (Thermo Scientific, Waltham, MA, USA) and ImageQuant LAS 4000 (GE Healthcare Biosciences AB, Uppsala, Sweden) with a chemiluminescent detection method. The exposure type was set to increment, sensitivity to high resolution and exposure time was every 30 s, for up to 15 min.

### 2.6 Immunoblot analysis

Signals from membranes were analyzed using VisionWorks® software (Analytik Jena GmbH, Jena, Germany). To describe the relative binding intensity of IgE-reactive proteins, the unit “band intensity” was used. The measured binding intensity was considered proportional to strength of antibody-binding to specific proteins. Based on that, the binding intensity of the different IgE-reactive protein bands was separated into five different binding categories: low, medium low, medium, medium high, and high. The obtained binding intensities were used to create allergograms, depicting each worker's possible immunologic sensitization to IgE-reactive proteins in the different salmon tissue extracts.

### 2.7 Mass spectrometry analysis

Protein bands corresponding to the molecular weight of IgE-binding proteins which had the most frequent binding pattern among salmon workers were cut out of an SDS-PAGE gel and analyzed using Liquid Chromatography-Mass Spectrometry (LC/MS). Mass spectrometric analysis was conducted at The Proteomics and Metabolomics Core Facility (PRiME) at the Department of Medical Biology, UiT The Arctic University of Norway as previously described (26). Proteins were filtered for an FDR of 0.01. Values that were used for the identification of proteins were coverage (%), number of peptides, number of peptide spectrum matches (PSMs), molecular weight (kDa), and accession number.

## 3 Results

### 3.1 Questionnaire responses

Study demographics and health outcomes based on questionnaire data are presented in Table 1. Of the 71 workers sensitized to salmon, 57 (80%) had answered the general questionnaire. Among those, 33/57 (58%) were male, with median age of 38 (range, 20–59) years. The median length of employment was 8 years, ranging from 1 to 23 years. Ever-smoking was reported from 35/57 (61%) of the workers. Any work-related symptoms were reported from 44/57 (77%) of the workers that answered the general questionnaire completely. Self-reported doctor-diagnosed asthma was established in 10/57 (18%) of the workers. Only 3 of the 57 responses (5%) indicated a self-reported allergy to salmon after ingestion.

**Table 1:** Demographics and health outcome of the study based on questionnaire data.

Demographics and health outcomes	Salmon processing workers, *N* = 57 *n* (%)
Gender	
Female	24 (42)
Male	33 (58)
Age years, median (range, min-max)	38 (20–59)
Employment years, median (range, min-max)	8 (1–23)
Ever smokers	35 (61)
Work-related symptoms during the past week	43 (77)
Eye	21 (37)
Nose	37 (65)
Lower respiratory	38 (67)
Self-reported doctor-diagnosed asthma	10 (18)
Self-reported allergy to salmon within two hours of ingestion	3 (5)

Percentages are based on the 57 workers that had answered the questionnaire completely, out of the 71 workers who were sensitized to salmon.

### 3.2 Sensitization to Salmon and common aeroallergens

Atopy was established in 36 of 69 (52%) workers whose serum was analyzed with ImmunoCAP, defined by ≥0.35 kU/L to at least one common seasonal IP6 or perennial IP7 allergen panel (Table 2). Elevated sIgE (≥0.1 kU/L) to salmon was identified in 30/69 (43%) of the sensitized workers. Eleven of the sensitized workers were recruited solely on sIgE to salmon with levels between 0.1 and 0.35 kU/L. A positive SPT (wheal diameter ≥3 mm larger than negative control) to at least one of the different extracts was confirmed in 56/71 (79%) of the workers. Most positive reactions were seen to the in-house raw muscle extract, followed by mucus, cooked muscle, and skin. For the commercial extracts, 8/71 (11%) and 6/71 (8%) were positive to cod and salmon, respectively. A total of 41/71 (58%) sensitized workers were included solely based on a positive SPT, whereas 14/69 (21%) workers were included by being sIgE positive to salmon exclusively. The prevalence of sensitization to salmon was found to be 7.3%, based on the inclusion criteria of a positive SPT (wheal diameter ≥3 mm larger than negative control) and/or positive sIgE (≥0.1 kU/L) to salmon, among the 977 examined workers.

**Table 2:** Allergic sensitization and serum diagnostics of the study population.

Sensitization outcomes	Salmon processing workers, *N* = 71 *n* (%)
Atopy^a^	36 (52)*
sIgE positive, salmon^b^	30 (43)*
Skin prick test positive^c^	56 (79)
*In-house extract* ^d^	
Raw muscle	43 (61)
Cooked muscle	12 (17)
Mucus	30 (42)
Skin	2 (3)
*Commercial extract* ^e^	
Cod	8 (11)
Salmon	6 (8)

^a^ IgE ≥0.35 kU/L (ImmunoCAP) to at least one of the two common inhalation panels: IP6 (birch, timothy, mugwort, *alternaria alternata*, *cladosporium herbarum*), and IP7 (cat, horse, dog, pteronyssinus, rabbit).

^b^ sIgE ≥0.1 kU/L (ImmunoCAP).

^c^ SPT wheal diameter ≥3 mm larger than negative control.

^d^ In-house salmon tissue extracts.

^e^ Commercially available SPT extracts.

* Percentage based on 69 workers whose serum were analyzed with the ImmunoCAP system.

### 3.3 Serum IgE-binding

**Figure 1:**
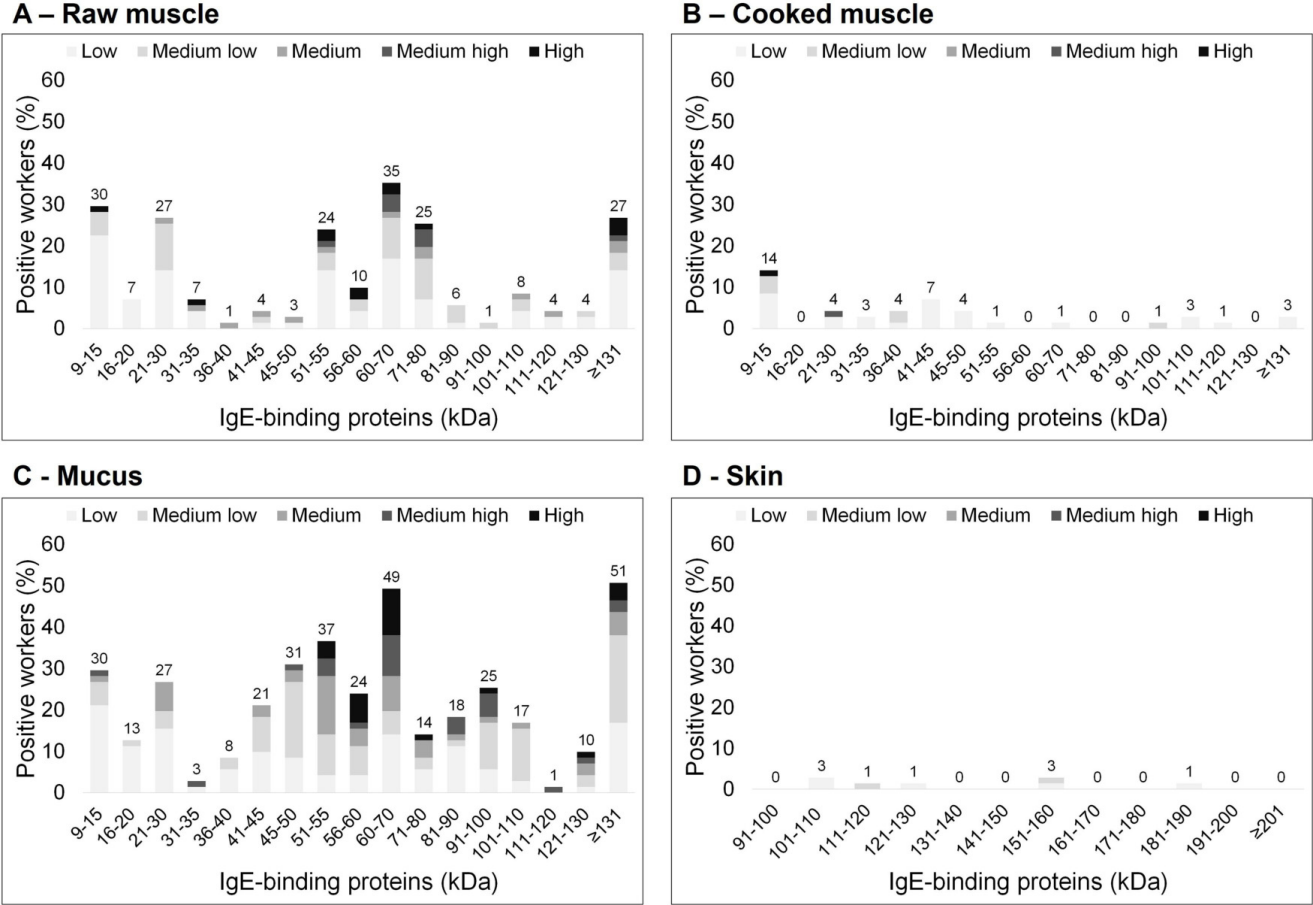
Stacked bar charts of serum IgE-binding proteins in sensitized Salmon processing workers (*n* = 71) using Salmon protein extracts from raw muscle **(A)**, cooked muscle **(B)**, mucus **(C)**, and skin **(D)** the *X*-axis represents the different regions of molecular weight (kDa), and the *Y*-axis is the percentage of workers with IgE-binding to Salmon proteins. The different shades represent the relative binding intensity to the different proteins, based on immunoblots with serum from workers, meaning that “low” is relatively weak band-intensity, and “high” is relatively strong band-intensity. The numbers above each bar represent the total percentage of workers having IgE-binding to that molecular weight area.

All 71 (100%) workers included in this analysis had IgE-binding to proteins in at least one of the tissue extracts, using immunoblot protocols (Table 3). In Figure 1, the results from immunoblotting are shown. In the raw muscle extract (Figure 1A), frequent binding was seen in regions of low molecular weight: 9–15 and 21–30 kDa, as well as relatively high molecular weight: 51–55, 60–80, and ≥131 kDa. With the cooked muscle extract (Figure 1B), only 14/71 (20%) of the workers had IgE-binding to any of the molecular weight regions, with the most frequent binding in the 9–15 kDa area. All 71 workers had IgE-binding to proteins in the mucus extract (Supplementary Figure S1), with most frequent binding occurring at protein bands between 60 and 70 kDa and ≥131 kDa (Figure 1C). Moreover, the strongest relative binding intensity was most commonly found in protein bands of 51–70 kDa. Overall, more workers displayed strong band intensities to any of the proteins in the mucus extract. Only 4/71 (6%) of the workers had IgE-binding to the skin extract, and this was observed at molecular weights >101 kDa (Figure 1D). The molecular weight areas which were most frequently involved in IgE-binding were further investigated along with the frequency of work-related symptoms among workers (Table 4). Work-related symptoms from nose and the lower respiratory system were most often reported by workers exhibiting IgE-binding to proteins in the 40–80 and >131 kDa areas in the mucus extract.

**Table 3:** IgE-binding to in-house and commercial protein extracts among sensitized workers using immunoblot protocols with in-house and commercial tissue extracts.

Immunoblot outcomes	Salmon processing workers, *N* = 71 *n* (%)
Immunoblot positive	71 (100)
*In-house extract* ^a^	
Raw muscle	56 (79)
Cooked muscle	14 (20)
Mucus	71 (100)
Skin	4 (6)
*Commercial extract* ^b^	
Cod	17 (24)
Salmon	0 (0)

^a^ In-house salmon tissue extracts.

^b^ Commercially available SPT extracts.

**Table 4:** Frequency of work-related symptoms among workers stratified by IgE-binding to Salmon proteins and molecular weight.

Molecular weight of proteins in extracts	Work-related symptoms, *n* (%)		
Raw muscle (kDa)	Eye	Nose	Lower respiratory
9–20	7 (12)	15 (26)	15 (26)
40–59	4 (7)	9 (16)	12 (21)
60–80	7 (12)	14 (25)	17 (30)
>131	3 (5)	8 (14)	11 (19)
Cooked muscle (kDa)			
9–20	3 (5)	7 (12)	6 (11)
40–50	2 (4)	6 (11)	7 (12)
60–80	0 (0)	1 (2)	1 (2)
>131	1 (2)	2 (4)	2 (4)
Mucus (kDa)			
9–20	7 (12)	8 (14)	13 (23)
40–59	16 (28)	32 (56)	33 (58)
60–80	9 (16)	15 (26)	20 (35)
>131	7 (12)	17 (30)	19 (33)
Skin (kDa)			
101–110	0 (0)	1 (2)	1 (2)
111–120	1 (2)	1 (2)	1 (2)
>131	1 (2)	2 (4)	2 (4)

*N* = 57 who answered the general questionnaire.

### 3.4 Monosensitivity to Salmon proteins

Following immunoblotting with serum from 71 workers sensitized to salmon, we identified monosensitivity to salmon proteins in 20/71 (28%) workers (Table 5). Two workers were monosensitized to proteins in the mucus extract, where one of them reported eye and respiratory symptoms; the other worker had not completed the questionnaire. Fourteen workers were monosensitized to proteins in the raw muscle extract, with five of those having binding to the 9–15 kDa area. One worker had binding to proteins in the 9–15 kDa area in both raw and cooked muscle extracts. A total of eleven workers that were monosensitized to proteins in the raw muscle extract also reported work-related symptoms, mostly nose symptoms. Four workers were monosensitive to proteins in the cooked muscle extract, where three had binding to the 9–15 kDa area. Three of the workers reported on work-related symptoms, mostly nose symptoms.

**Table 5:** Monosensitivity to Salmon proteins in mucus, raw and cooked muscle protein extracts among sensitized Salmon processing workers (*n* = 71), along with self-reported work-related symptoms during the past week.

Mucus	kDa	Work-related symptoms		
Worker no.		Eye	Nose	Lower respiratory
32	51–55	Yes	No	Yes
67	>131	N/A	N/A	N/A
*n* = 2		*n* = 1	*n* = 0	*n* = 1
Raw muscle	kDa	Work-related symptoms		
Worker no.		Eye	Nose	Lower respiratory
4	9–15	N/A	N/A	N/A
31	9–15	No	Yes	Yes
40	9–15	No	Yes	Yes
44	9–15	No	Yes	No
50	21–30	Yes	Yes	Yes
68	21–30	Yes	Yes	Yes
14	31–35	No	Yes	Yes
18	60–70	No	No	No
25	60–70	Yes	No	No
46	60–70	N/A	N/A	N/A
60	60–70	N/A	N/A	N/A
5	101–110	No	Yes	Yes
53	101–110	No	No	No
19	111–120	Yes	Yes	No
62	>131	Yes	Yes	Yes
*n* = 15		*n* = 5	*n* = 9	*n* = 7
Cooked muscle	kDa	Work-related symptoms		
Worker no.		Eye	Nose	Lower respiratory
33	9–15	Yes	Yes	Yes
44	9–15	No	Yes	No
54	9–15	No	No	No
13	51–55	No	Yes	No
*n* = 4		*n* = 1	*n* = 3	*n* = 1

N/A, did not answer questionnaire; n, number of workers with monosensitization and/or symptoms.

### 3.5 Mass spectrometric analysis

Protein bands which were most frequently involved in IgE-binding in serum from workers were considered relevant for further analysis. The bands were cut out of a protein gel (Supplementary Figure S2) and analyzed with mass spectrometry (Table 6). This confirmed the presence of the WHO/IUIS registered salmon allergens parvalbumin, enolase, aldolase, and collagen. Additionally, the analysis identified the presence of possibly novel allergens. Four bands were cut out from the gel lane containing the raw muscle extract. Here, parvalbumin was identified in the 12 kDa band, and triosephosphate isomerase in the 30 kDa band. The putative salmon allergens G-6-PI and pyruvate kinase were identified in the 60 and 70 kDa bands, respectively, where 70 kDa band most frequently caused IgE-binding with the raw muscle extract. From the cooked muscle extract, a 12 kDa band was cut out, which was found to contain parvalbumin. From the mucus extract, a total of 5 bands were cut out. Here, parvalbumin was also identified in the 12 kDa band. Both aldolase, GAPDH, and creatine kinase were identified in the 40 kDa band. Frequent IgE-binding was seen in the 55 kDa protein band in the mucus extract, where G-6-PI and enolase were identified. Frequent binding among workers was also seen to the 70 kDa mucus band, where pyruvate kinase was identified. One isoform of collagen was found in the 130 kDa mucus band, which 11 workers had IgE-binding to. Various isoforms of collagen were identified in the skin extract, with only one worker having binding to the 180 kDa band.

**Table 6:** WHO/IUIS registered Salmon allergens in protein extracts detected by mass spectrometry, from SDS-PAGE protein bands.

Raw muscle			Cooked muscle		
Mw (kDa)	Protein(s) in band	*n* workers with IgE-binding (%)	Mw (kDa)	Protein(s) in band	*n* workers with IgE-binding (%)
70	Pyruvate kinase^a^	34 (48)	12	Parvalbumin	10 (14)
60	Glucose-6-phosphate isomerase^a^	17 (24)			
30	Triosephosphate isomerase	19 (27)			
12	Parvalbumin	20 (28)			
Mucus			Skin		
Mw (kDa)	Protein(s) in band	*n* workers with IgE-binding (%)	Mw (kDa)	Protein(s) in band	*n* workers with IgE-binding (%)
130	Collagen alpha-1(I) chain	11 (16)	230	Collagen alpha-1(I) chain Collagen alpha-2(I) chain isoform X1	–
70	Pyruvate kinase^a^	35 (49)	200	Collagen alpha-1(I) chain Collagen alpha-2(I) chain isoform X1	–
55	Glucose-6-phosphate isomerase^a^ Enolase	42 (59)	180	Collagen alpha-2(I) chain isoform X1 Collagen alpha-1(I) chain-like	1 (1)
40	Aldolase Glyceraldehyde-3-phosphate dehydrogenase^a^ Creatine kinase	24 (34)			
12	Parvalbumin	21 (30)			

The identified proteins are presented corresponding to their approximate molecular weight; parvalbumin (12 kDa), enolase (47.3 kDa), aldolase (40 kDa), tropomyosin (37 kDa), collagen alpha (130–140 kDa), creatine kinase (43 kDa), triosephosphate isomerase (25 kDa), pyruvate kinase (65 kDa), glucose-6-phosphate isomerase (60 kDa), and glyceraldehyde-3-phosphate dehydrogenase (36 kDa).

^a^ Allergens not registered with WHO/IUIS but previously suggested as putative allergens in salmon (14).

## 4 Discussion

In the present study we aimed to investigate sensitization to salmon among occupationally exposed workers in the Norwegian salmon processing industry. A total of 977 workers were tested for sIgE to salmon (ImmunoCAP), as well as with SPT using both in-house and commercial protein extracts. The prevalence of salmon sensitization was found to be 7.3% (*n* = 71), based on either a positive SPT and/or elevated sIgE to salmon. Of the 71 workers with salmon sensitization, 57 (80%) completed the questionnaire on work-related symptoms. Among those, 44/57 (77%) reported on work-related symptoms from either eye, upper and/or lower respiratory system during the past week.

An important finding in this study was that the majority of workers were not sensitized to the major food allergen parvalbumin, demonstrated through few workers being SPT and immunoblot positive to the cooked muscle extract (Tables 2, 3). If parvalbumin was a major allergen causing sensitization in our study population, we would have expected to see more workers having a reaction to this extract, as parvalbumin is highly stable towards thermal treatment (8). Most workers were SPT positive to the raw muscle extract, followed by the mucus extract (Table 2). Moreover, all sensitized workers had IgE-binding to proteins in the in-house mucus extract, demonstrated through immunoblot protocols (Table 3). Interestingly, work-related symptoms were more frequently seen in workers exhibiting IgE-binding in the 40–80 and >131 kDa areas for mucus, than for raw muscle (Table 4).

Previous research has found IgE-binding to parvalbumin in 75%–100% of patients mainly exposed through ingestion (9, 18, 30). In contrast, our study population includes individuals who are exposed at their workplace, mainly to raw fish, and different types of tissues. In our study population, all workers had IgE-binding to the mucus extract with immunoblot protocols (Table 3), and 30/71 (42%) were SPT positive to the same extract (Table 2). In a previous publication, we reported on the absence of parvalbumin in our in-house mucus tissue extract (26). In the present study, however, we identified parvalbumin in the extract with mass spectrometry (Table 6). The protein content of the outer mucus coating of fish might vary depending on the surrounding environment of the fish, such as variations in stress factors (31, 32). The salmon that we used for extraction in the referred paper came from a research facility where the salmon was kept in tanks, with no extensive slaughtering processes being performed. In contrast, the salmon that was used for making the protein extracts in the current paper was obtained from a salmon processing plant. Moreover, the salmon was also infected with sea lice upon arrival at our lab. Considering that only 21/71 (30%) of the workers had IgE-binding to the 9–15 kDa area with immunoblot protocols and the mucus extract (Figure 1C), and that the number of workers that reacted to the cooked muscle extract, with either SPT or the immunoblot assay, was relatively low, we consider parvalbumin to be a minor allergen causing sensitization in this study population of occupationally exposed salmon processing workers.

Frequent IgE-binding to the 40–55 kDa area was seen with the mucus extract and immunoblot protocols (Figure 1C). With mass spectrometry, we identified the presence of heat-labile allergens in mucus in these molecular weight areas (Table 6). Interestingly, the molecular weight region in which the sensitized workers had most frequent binding to, and which most often caused the strongest band intensity, was the 60–70 kDa region in the mucus extract (Figure 1C). This indicates binding to the putative salmon allergens pyruvate kinase and G-6-PI (14), two proteins that we detected both in the raw muscle and mucus protein extracts with mass spectrometry (Table 6). The presence of G-6-PI was verified in the in-house mucus extract, using commercial antibodies (data not shown). Previous studies on fish allergy have mostly focused on exposure to fish muscle (33–36). However, allergens from fish have been identified in other sources, such as fish roe (37), skin (17, 38), and fish blood (39). With the data that we have presented here on the prevalence of positive test results with the in-house mucus extract, using SPT and immunoblot assays, we also suggest that the outer mucus coating of salmon might be an important source of allergens in the salmon processing industry.

When investigating the relationship between the prevalence of work-related airway and eye symptoms and IgE-binding proteins (Table 4), we found the highest symptom frequency among workers with IgE-binding to mucus proteins in the 40–59 kDa range, followed by those with IgE binding to 60–80 and >131 kDa proteins in the same extract. Pyruvate kinase has previously been described as a putative allergen in cases of shrimp and crab allergy (40) as well as swordfish allergy (41). G-6-PI is exclusively registered as a catfish allergen in the WHO/IUIS database. To our knowledge, there are no studies describing either of the two putative allergens as potential sensitizers in the occupational setting, such as the seafood processing industry. The proteins were not submitted to the WHO/IUIS database, as more research is needed to sufficiently separate the proteins from each other.

We identified 2/71 (3%) of the workers as SPT positive to the skin extract (Table 2), and 4/71 (6%) showed IgE-reactivity to proteins in the skin extract with the immunoblot assay (Table 3). Proteomics analysis identified various isoforms of collagen in the skin tissue extract (Table 6). Collagen is non-soluble in neutral aqueous solutions, which might explain why this protein is underrepresented in commercially available SPT extracts (42). A previous study comprising fish-allergic patients from Hong Kong and Japan reported a 38.9% sensitization rate to collagen (18) whereas the study where collagen was first described as an allergen reported a sensitization rate of 21% among patients with a previous history of dietary allergy to fish (17). The latter study involved patients from Australia, thus the differences in sensitization rates might be explained by varying dietary habits in different geographical locations.

With commercial SPT extracts from cod and salmon, we only identified 8/71 (11%) and 6/71 (8%) of the sensitized workers to be SPT positive, respectively (Table 2). Moreover, 17/71 (24%) of the sensitized workers had IgE-binding to the cod extract with immunoblot protocols, whereas none had IgE-binding to the commercial salmon extract (Table 3). Coomassie staining of a protein gel revealed little to no visible bands in the lane containing this extract (Supplementary Figure S2). We did not investigate the commercial extracts any further with mass spectrometry; however, previous studies have described the varying presence of allergens in commercially available SPT extracts (42, 43). The discrepancy of SPT-results when using our in-house extracts compared to using the commercial cod and salmon extracts, further emphasizes the importance of having well-characterized SPT extracts containing allergens that are relevant in both dietary- and occupational allergies available for diagnosis.

It is well documented that atopy is a risk factor for IgE-mediated sensitization to high molecular weight allergens in occupational settings (44). The atopy status of the workers could therefore influence the outcome in our study population. We found that 36/69 (52%) of the sensitized workers were atopic, defined by IgE ≥0.35 kU/L to either IP6 and/or IP7 (ImmunoCAP) (Table 2). The 0.35 kU/L cut-off was chosen to ensure a low risk of false-positives and allow comparison with other relevant studies. Sensitized workers to be investigated further by immunoblot test protocols were selected based on either a positive SPT and/or elevated sIgE to salmon. The cut-off level for sIgE to salmon was set at 0.1 kU/L, a threshold that is increasingly referred to in literature (45, 46). It is based on the improved sensitivity and reproducibility offered by newer laboratory test systems, combined with the recognition that low levels of sIgE can be clinically relevant in some individuals. The rationale for choosing this lower cutoff level in our study was to ensure that individuals with low yet clinically significant responses were not excluded, as the clinical impact of low-level exposure has not previously been examined in salmon worker populations. We found that 11/69 (16%) individuals in the study population had sIgE to salmon between 0.1 and 0.35 kU/L. Seven of the eleven individuals (64%) reported work-related eye and/or upper/lower respiratory symptoms during the past week. This indicates that lower-range sIgE-levels may be clinically relevant for at least some workers in the salmon industry, possibly representing an initial allergic response.

With the ImmunoCAP system, we found that 30/69 (43%) of the workers had elevated sIgE to salmon (Table 2). On the other hand, when investigated with immunoblot, all workers in our study population, including those with sIgE levels between 0.1 and 0.35 kU/L, had detectable IgE-binding to proteins in at least one of the in-house produced tissue extracts (Table 3). Fifty-six of the 71 workers were SPT positive (79%) (Table 2). We therefore suggest that our in-house tissue extracts are better diagnostic tools for detecting allergic sensitization in salmon processing workers, than the commercially available extracts and test systems. Previous studies have found the ImmunoCAP assay to have relatively low sensitivity for the presence of serum IgE-binding proteins, possibly due to an under-representation of heat-labile proteins (14, 47). The potential lack of heat-labile proteins in the commercial salmon extracts used in the present study is especially relevant in the occupational setting of the seafood processing industry, as the examined workers are exposed to raw fish.

In the present study, we investigated the possible relationship between monosensitivity to salmon allergens and work-related symptoms among workers sensitized to salmon (Table 5). Monosensitivity to proteins was most frequently observed to the raw muscle extract (*n* = 15). Ten of those fifteen also reported work-related symptoms, mostly respiratory symptoms. Four workers were monosensitive to proteins in the cooked muscle extract, and two to the mucus extract. In addition, four workers were monosensitive to raw muscle in the 60–70 kDa area (Table 5). With mass spectrometry we identified the presence of the previously described putative allergens pyruvate kinase and G-6-PI (14), suggesting that they could act as sensitizers in the occupational context. Cases of monosensitivity to other fish allergens than parvalbumin have previously been described (40, 41, 48). More particularly, the latter study described three patients who were sensitized to the heat-labile fish allergens aldolase and enolase but were negative to parvalbumin (48). More research is needed to assess the clinical relevance of monosensitization to different allergenic salmon proteins.

The prevalence of salmon sensitization in our study population was found to be 7.3% (*n* = 71). Previously reported prevalences of sensitization to salmon in the salmon industry varies from 0.0% to 8.6% (4, 5, 49, 50). In a recent case series, salmon-processing workers with occupational asthma were found to be sensitized to salmon muscle, skin, and intestines (51). Among the sensitized workers in our study, 57 (80%) had completed the questionnaire, including questions on specific symptoms during the workday the last week. Nose and lower respiratory symptoms were most prevalent, reported by 37/57 (65%) and 38/57 (68%), respectively. Other publications from the SHInE study are planned, which will include data on asthma and symptoms, combined with sensitization among the workers.

In clinical settings, conclusions on occupational allergic disease may often rely on a positive sensitization test, such as the ImmunoCAP or SPT. Using these approaches there is a risk of underdiagnosis of disease. Eventually such underdiagnosis might lead to chronification of the disease following continued exposure. If the diagnostic tools are weak, negative test outcomes used to exclude diagnosis of e.g., occupational asthma, may lead to falsely denial of legitimate claims for worker compensation. Hence, it is important to have test extracts containing a panel of allergens that are representative for the actual workplace exposure the workers are experiencing. Moreover, prevention of disease development and possibly exacerbation of work-related symptoms is possible through increased knowledge about which processes, tissues and proteins are most often involved in sensitization.

The data presented in this study was collected as part of a cross-sectional study and is thus prone to selection bias. Workers already experiencing health issues might be more eager to participate, thus increasing the prevalence of sensitization in the study population. On the other hand, a potential healthy worker effect might be present, whereby individuals who previously experienced severe work-related symptoms may have left the industry prior to data collection and, consequently, are not represented in the study population. This can lead to an underestimation of the prevalence of sensitization. The use of a questionnaire inherently carries a risk of recall bias among participants; however, this concern is mitigated in the present study by the short recall period, limited to the past week. A strength of the study is the use of in-house salmon tissue extracts in both skin prick testing, and the detailed analysis of individual IgE-binding profiles with immunoblot protocols. The extracts contained IgE-binding proteins of relevance to the occupational exposure situation in the salmon processing industry. Another strength is the relatively large number of involved participants, and in particular the extensive testing with immunoblot protocols for identification of IgE-reactive proteins.

## 5 Conclusion

In this study population of 977 employees in the Norwegian salmon industry, we identified a prevalence of immunologic sensitization to salmon of 7.3%. Known and putative allergenic proteins were identified based on immunoblots of workers’ serum with salmon tissue extracts. Based on the finding that 100% of the sensitized workers had IgE-binding to proteins in the in-house mucus extract, we suggest that contact with salmon mucus during salmon processing is associated with a high risk of sensitization, as is contact with raw salmon muscle. Proteins in the 60–70 kDa area seem to be strong sensitizers and includes pyruvate kinase and glucose-6-phosphate isomerase, which are proteins not previously identified as allergens in salmon. Further studies should aim to characterize the IgE-binding capacity of these proteins as well as the association with allergy symptom development. We suggest that other allergens than parvalbumin are important as sensitizers in occupationally exposed salmon workers. This underlines the importance of having diagnostic tools that cover a variety of allergens present in different types of exposure situations.

## Data Availability

The original contributions presented in the study are publicly available. This data can be found here: https://dataverse.no/dataset.xhtml?persistentId=doi:10.18710/YUJPJT.

## Supplementary material

SUPPLEMENTARY FIGURE 1 Immunoblot results from 71 sensitized salmon processing workers.

SUPPLEMENTARY FIGURE 2 SDS-PAGE and Coomassie staining of skin prick test extracts. 1: commercial cod, 2: commercial salmon, 3: in-house raw muscle, 4: in-house cooked muscle, 5: in-house mucus, and 6: in-house skin.
