# Supplementary figures and images for: Sensitization to salmon among occupationally exposed Norwegian salmon processing workers: identification of IgE-reactive proteins
Source: Front Allergy. 2026 Feb 16;7:1735903. doi: 10.3389/falgy.2026.1735903 (PMC12950587; doi:10.3389/falgy.2026.1735903)

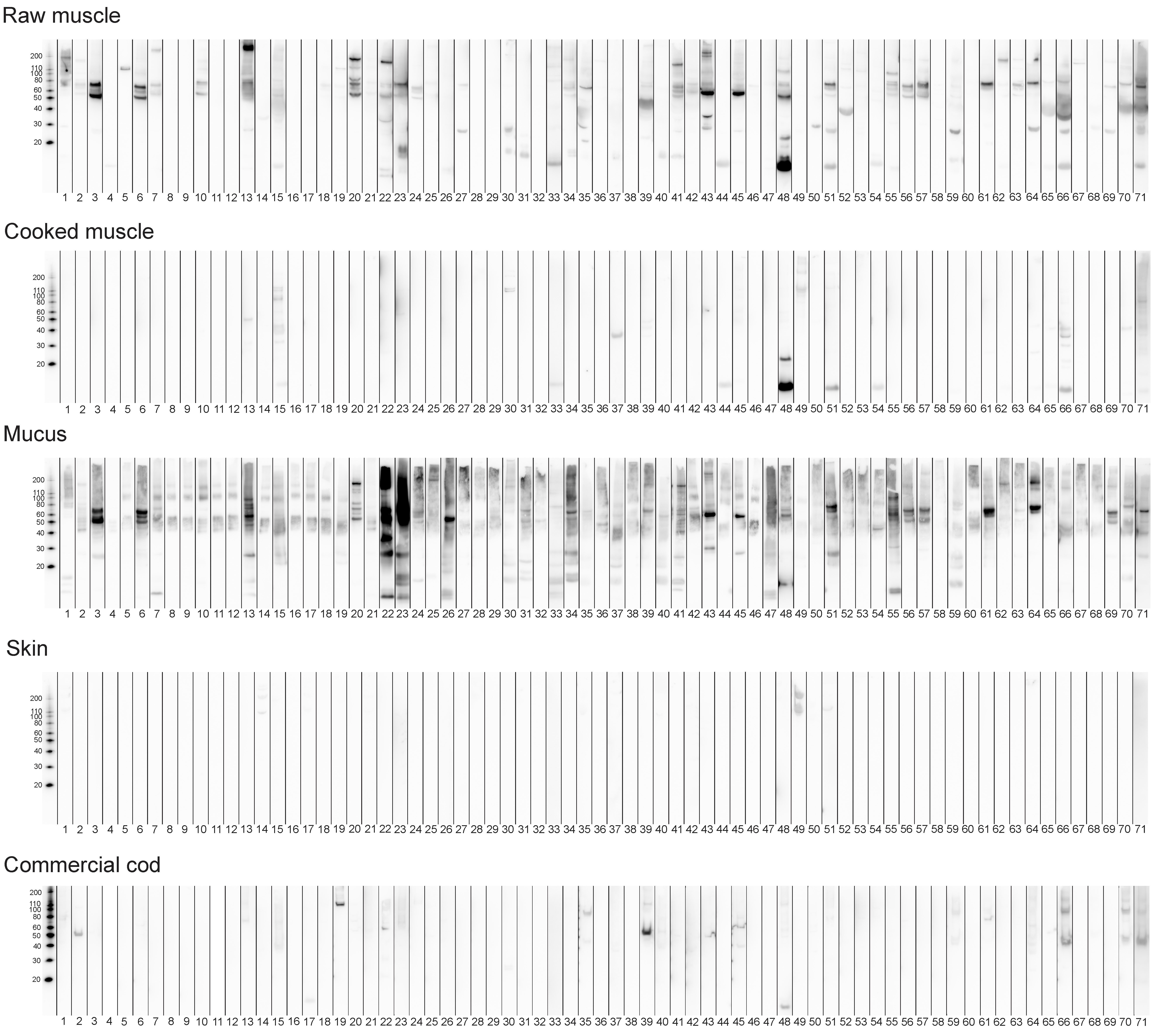

Supplement: SUPPLEMENTARY FIGURE 1 — Immunoblot results from 71 sensitized salmon processing workers. [file Image1.jpeg]

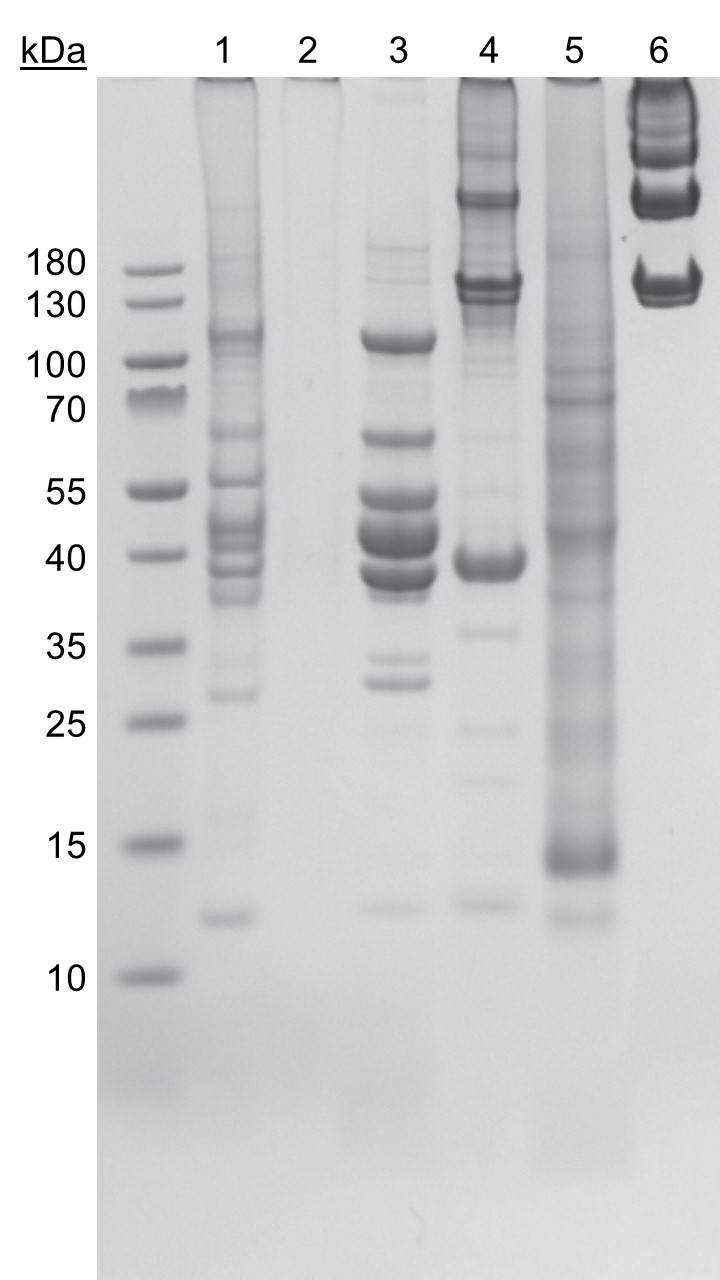

Supplement: SUPPLEMENTARY FIGURE 2 — SDS-PAGE and Coomassie staining of skin prick test extracts. 1: commercial cod, 2: commercial salmon, 3: in-house raw muscle, 4: in-house cooked muscle, 5: in-house mucus, and 6: in-house skin. [file Image2.jpeg]
